# Emerging Nucleic Acid–Based Tests for Point-of-Care Detection of Malaria

**DOI:** 10.4269/ajtmh.2012.11-0685

**Published:** 2012-08-01

**Authors:** Michael S. Cordray, Rebecca R. Richards-Kortum

**Affiliations:** Department of Bioengineering, Rice University, Houston, Texas

## Abstract

Malaria remains a serious disease in the developing world. There is a growing consensus that new diagnostics are needed in low-resource settings. The ideal malaria diagnostic should be able to speciate; measure parasitemia; low-cost, quick, and simple to use; and capable of detecting low-level infections. A promising development are nucleic acid tests (NATs) for the diagnosis of malaria, which are well suited for point-of-care use because of their ability to detect low-level infections and speciate, and because they have high sensitivity and specificity. The greatest barrier to NAT use in the past has been its relatively high cost, and the amount of infrastructure required in the form of equipment, stable power, and reagent storage. This review describes recent developments to decrease the cost and run time, and increase the ease of use of NAT while maintaining their high sensitivity and specificity and low limit of detection at the point-of-care.

## Introduction

Malaria is one of the most serious of the diseases of poverty and is widespread across the developing world causing 200–500 million illnesses, and more than 1 million deaths each year.^1^–^3^ Malaria accounts for up to 20% of all childhood deaths in Africa.^4^–^6^ In many of the areas to which malaria is endemic, there is a lack of access to effective diagnostics, leading to poor surveillance of malaria infections and treatment, poor health outcomes for non-malarial fever patients, and a public lack of trust in the health system.^7^ In addition, over-prescription of cheap antimalarial drugs has led to development of widespread drug resistance, and drugs that remain effective, such as artemisinin-derived treatments, are much more expensive (approximately $1–$2 per day), leading to an increased need for accurate diagnosis before treatment.^8^–^12^ Thus, there is a growing consensus that there is a need for new diagnostics that are more accessible in malaria-endemic areas, and that have improved performance over existing techniques to help guide the distribution of antimalarial drugs, to more effectively target the disease, and to reduce the generation of drug-resistant strains.^13^

A major limitation of malaria diagnosis is that most cases occur in areas with limited health care infrastructure.^3^,^14^–^16^ To be useful in these circumstances, a diagnostic should be low cost, require minimal or no external power, be able to be run on portable and easy to maintain equipment, be usable without extensive training, not require refrigerated reagent storage and deliver accurate and unambiguous results rapidly. The World Health Organization has established a set of principles to guide the development of diagnostics for these low-resource, point-of-care (POC) settings known as ASSURED. A test should be Affordable, Sensitive, Specific, User-friendly, Rapid and Robust, Equipment-free, and Deliverable to end-users.^17^ In addition, the ideal malaria diagnostic should be able to determine which species is infecting the patient, determine the level of infection (measured as parasitemia, the percentage of infected erythrocytes), and be capable of detecting low level infections.

Nucleic acid tests (NATs) have been suggested as a way to meet these criteria, and a number of NATs are being developed to detect malaria in POC settings. Currently, NATs for malaria are used primarily in central health facilities because they tend to be more resource intensive. The focus of this review is on the development of NATs that can be implemented in POC settings. For the foreseeable future, many of these NATs will be most practical in laboratory settings in these low-resource countries, but there is increased focus on adapting NATs into systems that might be directly used at the POC. In this article, we review current malaria diagnostics and discuss their benefits and drawbacks. We then examine emerging malaria NATs for POC settings, comparing their diagnostic performance and their potential utility in low-resource settings.

## Current Malaria Diagnosis

### Blood film microscopy.

The most commonly used laboratory diagnostic method for malaria is Giemsa-stained blood microscopy.^18^,^19^ A blood smear sample can be read by a skilled technician in 20 minutes and costs approximately $0.20 per sample, including the cost of staining reagents and the technician's time. Two different staining techniques, the thin and thick blood smear, are used for malaria diagnosis. The thin blood smear more accurately preserves malaria parasite morphology and enables easier speciation of the infecting parasite. A thick blood smear is used to quickly observe a larger volume of blood more quickly, which increases the sensitivity of the diagnosis by approximately 10-fold; however, it is more difficult to speciate the infection because of distortions in morphology.^20^ A highly trained technician using these techniques can reliably detect as few as 50 parasites/μL of blood, with a sensitivity and specificity of 95% and 98% (using the polymerase chain reaction [PCR] as a gold standard).^21^

There is a growing consensus that blood smear microscopy is inadequate as a malaria diagnostic. The quality of blood smear examination is highly dependent on the quality of the microscope, the quality of the available staining reagents, and the skill of the technician.^22^,^23^ A study comparing the performance of thick and thin blood smear microscopy in expert laboratory and field conditions tested 3,004 blood smears and found that the sensitivity decreased to 10% when samples were examined in rural villages in Thailand compared with the same samples reexamined in a laboratory.^23^ This change was attributed mainly to the difficulty less-trained technicians had recognizing low-level infections, and other research has found that microscopy may miss as many as 50% of the cases detected by PCR.^23^,^24^

### Antigen detection and rapid diagnostic tests.

A recent advance in malaria diagnosis has been the commercialization of lateral flow immunoassay rapid diagnostic tests (RDTs). These tests are easy to use because the user only needs to spot the patient's blood onto the base of the strip and wait for the result (approximately 20 minutes).^25^,^26^ The RDTs have a cost between $0.45 and $1.40 per test to the end user, and an average cost of approximately $0.85.^12^ Approximately 20% of the final cost of RDTs to the user represent shipping and local storage and transportation costs.^27^ The RDTs have a reported detection limit of > 100 parasites/μL and 80 –95% sensitivity and 85% specificity using microscopy as a gold standard.^12^,^26^,^28^–^34^

The World Health Organization recently launched a large review of the performance of commercially available malaria RDTs and found wide variation between tests from different manufacturers and between different lots of the same diagnostic. Variation was found to be especially high when parasitemia was less than 200 parasites/μL of blood.^35^–^37^ Recent studies have shown that there is greater variation than believed in the incidence and structure of the *Plasmodium falciparum* histidine-rich protein, which is the target of many RDTs, and might account for some of this variability.^38^–^40^ In addition, RDTs become degraded and less sensitive and specific at temperatures commonly found in POC clinics.^41^ Careful management of RDT procurement, transportation, and storage can prevent RDT degradation and they can be an effective tool in malaria diagnosis, but good management of RDTs increases the cost and difficulty of using them in POC environments.^42^ The RDTs can also show false-positive results because of antigens circulating for up to two weeks after the infection has ended.^43^ Malaria RDTs also do not currently offer the ability to test for the markers of drug resistance or to quantify the level of infection.^44^

### Polymerase chain reaction.

The NATs offer advantages because they can speciate infections and test for drug resistance.^43^,^45^–^49^ Most NATs for malaria focus on the 18S ribosomal RNA gene, which contains regions conserved across all *Plasmodium* species and regions specific to each species.^46^,^47^,^50^ Depending on the technology used, tests may target either the 18S gene directly or its associated mRNA. DNA targets offer the advantage of being more stable, enabling long-term storage of patient samples before testing. DNA circulating post-infection may lead to false-positive diagnostic results.^51^ The advantage of using RNA as a target for diagnostics is that it is much more abundant in the cell than DNA, with up to 1,000 rRNA copies of the 18S gene per parasite.^52^

The PCR is more sensitive than either RDTs or microscopy, and has been found to be especially effective at identifying low-level infections often missed by other techniques, and has a limit of detection of 0.5–5 parasites/μL.^53^–^57^ However, PCR- based assays are the least feasible to perform at the POC. The PCR is prone to contamination, the nucleic acids must be extracted and purified from the patient sample, and reagents must be stored cold to maintain their function. The reagents for PCR diagnostics can cost approximately $1.50 –$4 per test.^58^–^61^ A summary of the performance of current malaria diagnostics is shown in Table 1. There is a growing consensus that there is a need for new malaria diagnostic tools to overcome their limitations, especially in POC settings.^3^,^62^,^63^

## Emerging Nucleic Acid Tests

The NATs have the potential to offer many advantages at the POC, such as low limits of detection, the ability to speciate, and to quantify the level of infection. For malaria diagnosis, many NATs consist of a separate amplification and detection step because there is insufficient *Plasmodium* nucleic acid in a peripheral blood sample for direct detection by using current detection technologies.^47^ Emerging NATs for malaria diagnosis seek to be appropriate for the POC through a variety of methods, including reducing the cost and difficulty of the amplification step and generating a quick and easy to use detection schemes. The diagnostic performance of the POC NATs discussed below is summarized in Table 2.

### Isothermal amplification.

Isothermal amplification techniques operate at a single temperature, eliminating the need for a thermocycler, enabling them to be conducted on simple and portable heating systems. Several isothermal amplification techniques have been developed in recent years, such as helicase-dependent amplification, rolling circle amplification, and nicking enzyme amplification reaction.^64^–^66^ Of the various isothermal amplification techniques, two in particular, loop-mediated isothermal amplification (LAMP) and nucleic acid sequence–based amplification (NASBA), have been extensively explored for malaria diagnosis.

#### Loop-mediated isothermal amplification.

The most extensively studied amplification technique for the detection of malaria is LAMP. This procedure uses a complex set of four primers that after initial binding and amplification steps form a stem-and-loop structure, which leaves a binding site constantly open for new primers to anneal. The LAMP is a highly efficient technique capable of achieving 10^8^ fold amplification in as little as one hour.^67^ It also generates a magnesium pyrophosphate precipitate during amplification, which can be used for detection by measuring the turbidity of the solution.^68^,^69^

Loop-mediated isothermal amplification was first used for diagnosis of malaria in 2006 for n = 202 patient samples obtained in Thailand. An easy to implement sample preparation method was also investigated during this study, which was boiling blood samples of the patients for 10 minutes to release the parasite DNA. The amplified malaria DNA was monitored by using a real-time turbidity measurement to track generation of a precipitate. This study used microscopy as a gold standard and determined that LAMP had a sensitivity of 95% and a specificity of 99%. Investigators detected 10 copies of target serially diluted from a clinical specimen in a 50 μL sample, suggesting a detection limit of 0.2 parasites/μL of blood.^70^

The LAMP primer sets have been designed for each of the four species of malaria, as well as a pan-*Plasmodium* set. These LAMP primers were tested on 121 patient samples obtained in rural Thailand. DNA preparation was accomplished by boiling the blood samples for 10 minutes. The pan-specific reactions provided results in approximately 25 minutes, and each of the species-specific amplifications provided results in approximately 35 minutes. The limit of detection was determined by using positive control plasmids; for *P. malariae* and *P. ovale*, this limit of detection was 0.2 parasites/μL of patient blood, and the limit of detection for remaining primer sets was 2 parasites/μL of blood. Using microscopy as a gold standard, the investigators found that on average for each of the primer sets, the sensitivity was 98.5% and the specificity was 94.3%.^71^

The performance of LAMP using turbidity for detection and boiling for DNA extraction was compared with gel electrophoresis and a commercial DNA extraction kit on samples from Bangladesh (n = 115). Investigators found that LAMP using commercial DNA extraction and gel electrophoresis had a sensitivity of 76.1% and a specificity of 89.6% (laboratory PCR was used as a gold standard). Although boiling alone did not affect the performance of the assay compared with that of the commercial kit, using boiling and turbidity as a detection method together decreased the specificity to 58.3%. This decrease in specificity was attributed to the non-specific nature of visual detection, and the increase in non-targeted DNA from the sample preparation technique. The investigators in this study concluded that further optimization of LAMP to reduce non-specific amplification is necessary before heating can be used as a POC sample preparation technique, but that overall LAMP remains a promising technique for malaria diagnosis. The reagent cost of LAMP was $0.40 –$0.70 per test, which was comparable to the price to the consumer of an RDT strip.^72^ However, extra LAMP reactions would need to be run as positive controls, which are included in the price of a single RDT, increasing the cost of LAMP. This cost is also increased by the additional resources required to implement it.

A heating system has been developed for LAMP that does not require an external power source. The system uses an exothermic reaction, CaO and water, and the heat from this reaction is coupled with a phase-change material with a melting temperature of 62°C. A prototype was built that maintained the LAMP amplification temperature (62– 65°C) for 45 minutes from a single CaO reaction. When turbidity was used as a detection method, LAMP could be conducted with samples spiked with *P. falciparum* DNA without requiring an external power source, maintaining the same limit of detection as LAMP conducted with a conventional laboratory heat source.^73^

The LAMP is a promising method for the POC diagnosis of malaria. It has a low limit of detection (0.2–2 parasites/μL), a high sensitivity and specificity, ranging from sensitivity = 76.1–98.5% and specificity = 89.6 –100%, produces a result in 30 minutes to 2 hours, and enables visual readout of results.^70^–^72^,^74^ One major drawback of LAMP is that it is prone to contamination and amplification of non-targeted DNA sequences, which decreases the specificity of the assay.^71^,^74^ There is a potential to increase the field specificity of LAMP by coupling it with a targeted detection system.^72^

#### Nucleic acid sequence–based amplification.

Nucleic acid sequence–based amplification is a different isothermal amplification method that has recently been applied to malaria diagnosis. The NASBA reaction continually cycles between the activity of a reverse transcriptase to copy an RNA sequence into a cDNA, and the activity of a T7 RNA polymerase for subsequent amplification. It generates a high number of RNA copies per cycle, enabling it to generate detectible product in a shorter time frame than other amplification techniques.^75^

The NASBA RNA probes were designed for the *P. falciparum* and *P. vivax* 18S mRNA and tested on 99 samples.^76^ The samples were amplified for one hour by using a commercial thermal cycler, which was used to monitor the progress of the NASBA reaction in real time. The limit of detection of the assay was determined by using serial dilutions of clinical samples and was 0.1– 0.01 parasites/μL of blood, and NASBA was found to be quantitative.^76^

The NASBA was further tested on samples from areas of high (rural Kenya, n = 149) and low (urban Tanzania, n = 154) malaria prevalence. The NASBA reaction was conducted in an real time PCR system alongside laboratory PCR with microscopy as a gold standard. In the sample set from Kenya, the correlation between NASBA and microscopy was 0.80, and PCR and microscopy had a correlation of 0.76. In the lower incidence population of Tanzania, the correlation of NASBA with microscopy decreased to 0.33, and the correlation of PCR with microscopy decreased to 0.25.^45^ Because PCR and NASBA are more sensitive to low-level infections, the decrease in correlation is caused by infections missed by microscopy, further suggesting that PCR and NASBA are especially useful in low prevalence areas where low-level infections are more common.^45^,^77^

The NASBA has a high degree of specificity and sensitivity, can produce results in an hour, has the lowest limit of detection of any of the investigated malaria diagnostics, and has a detection limit of 0.01 parasites/μL of blood as determined by serial dilution of clinical samples of known parasitemia. Some of the limitations of NASBA as a POC technique are that it is prone to contamination and false-positive results, and that it requires more extensive sample preparation than LAMP. The cost of NASBA reagents is approximately $5–$20 per test, making it expensive for malaria diagnosis.^78^ However, because it is the most sensitive to low-level infections, NASBA has the potential to be especially useful as a screening tool despite a relatively high cost.

Isothermal amplification techniques such as NASBA and LAMP are promising for malaria diagnosis in POC settings. They eliminate the need for a costly and power-intensive thermocycler; produce results in a short time, from 30 minutes (LAMP) to 1 hour (NASBA); and are capable of detecting infections of < 1 parasite/μL of blood. The sensitivity and specificity of these techniques are comparable to those of PCR-based diagnostics, although the specificity of LAMP decreases because of non-targeted amplification. The LAMP can also be used with samples that have undergone minimal pre-processing, and results can be monitored with turbidity measurements, making it especially suited for use as a malaria POC diagnostic because it has comparable material costs to the commercial price to users of RDTs.^72^ However, extra amplifications would be needed to be run to compensate for the fact that amplifications do not include a built-in positive control as in commercial RDTs. Although it is the most sensitive to low level infections, NASBA is currently too expensive for practical POC use, making it more appropriate for use in regional or central health facilities. One of the major drawbacks of isothermal amplification techniques is that so far they have been tested only in relatively well equipped laboratories.

### Novel PCR-based detection methods.

Although traditional PCR is an extremely sensitive (97%) and specific (100%) technique compared with microscopy, with the ability to amplify low levels of infection, it is limited at the POC by a susceptibility to contamination, expensive reagents, and the need for a stable power supply and thermocycler.^79^–^81^ There is a growing interest in developing novel processing and detection techniques to adapt laboratory PCR into a POC tool.

Several NATs for malaria at the POC focus on finding ways to couple PCR with novel detection systems. Novel detection strategies can be used to increase the utility of an amplification reaction by speciating malaria in a single reaction (one-pot detection), by reducing the amount of required amplification, or by removing the need for special detection systems.^68^,^69^,^80^,^82^,^83^ Some of these detection techniques may eventually be coupled with isothermal amplification techniques to combine the benefits of POC amplification and detection.

A commercial wellplate enzyme-linked immunosorbent assay (ELISA) has been adapted into a NAT for detection of malaria by using capture oligonucleotides instead of antibodies. Traditional PCR amplification biotinylated primers and a horseradish peroxidase–conjugated streptavidin are used with a chromogenic horseradish peroxidase substrate for detection. This technique was tested in the field on 300 patients in Thailand, where researchers were able to achieve a sensitivity of 91.4% and a specificity of 95.8% (using microscopy as a gold standard) and a detection limit of 30 parasites/μL, determined by the lowest microscopic parasitemia detected by the PCR-ELISA.^84^ The PCR-ELISA enables high throughput screening, is able to detect mixed infections, and has successfully been tested in a low-resource setting. However, the major drawback is that it requires the infrastructure to support PCR and a wellplate reader.

A major focus of research into adapting PCR for the POC is the development of single-pot detection systems that enable identification of more than one infecting species of malaria from a single amplification reaction. In the PCR ligase detection reaction (PCR LDR), DNA probes for each *Plasmodium* species are designed of different lengths to be resolved separately by electrophoresis on a gel.^80^ When tested with 189 samples from Papua New Guinea, the PCR LDR had a limit of detection using diluted cultured parasites of 1 parasite/μL, a sensitivity of 100%, and a specificity of 90%, using expert microscopy as a gold standard.^80^ Although this technique was able to detect each species of malaria from a single amplification, it is unsuited for POC use because it can take hours to resolve the different bands, requires a stable power supply to run the gel, and requires a secondary detection step to locate the DNA in the gel.

The PCR LDR was improved by introducing a fluorescent microsphere assay for detection (LDR-FMA). Commercial microspheres tagged with a specific sequence (Luminex FlexMap probes) are used for detection. Different FlexMap microspheres, uniquely labeled with specific fluorophores, recognize species-specific DNA sequences and are used to quantify the presence of individual *Plasmodium* species.^82^

The LDR-FMA was tested on cultured malaria samples and had a concordance > 90% with microscopy and a limit of detection of 20 parasites/μL of blood, which was determined on the basis of the lowest parasitemia measured by microscopy that was positive by LDR-FMA. The detection step was estimated to cost $0.30 per patient; however, this cost does not include the cost of the necessary amplification step.^82^ The drawback to this technique is that it is relatively time-consuming and requires a sophisticated BioPlex Array reader capable of measuring the difference between different fluorescent tags used.

Another approach to developing a fluorescent one-pot detection system for malaria used a series of fluorescence resonance energy transfer–based DNA probes and the commercial LightCycler system. Probes are designed to bind to a species-specific region of the 18S ribosomal DNA and each different probe was designed so that it had a unique melting temperature. By using a carefully controlled temperature source (an RT-PCR machine), it was possible to differentiate between different probes by measuring their melting temperatures. This method was tested on 297 samples from Thailand and enabled highly sensitive differentiation and separate quantitation of mixed parasite infections in a single reaction.^79^ When this technique used microscopy as the gold standard, it had a sensitivity of 97% and a specificity of 100%. The limit of detection was determined by using a positive control plasmid spiked into uninfected blood samples and was 1 parasite/μL. The major drawback of this technique as a POC tool is that it requires sophisticated temperature control and fluorescence detection systems.

The nucleic acid lateral flow immunoassay (NALFIA) is an attempt to create a rapid, easy-to-use detection method for DNA targets that is entirely self-contained. The NALFIA is intended to be coupled with an isothermal amplification method, although so far it has only been tested with laboratory PCR. The NALFIA is analogous to the lateral flow immunoassay technology used in RDTs, but uses DNA capture and recognition sequences and antibodies to attach these sequences to the reporter molecule and the nitrocellulose. As with RDTs, the reagents can be pre-set on the nitrocellulose strip so that a user only needs to apply a sample and wait for a result to develop (approximately 10 minutes).^85^

The NALFIA was tested under field conditions in Mbita, Kenya on 650 patient samples. Samples were purified by using commercial kits and amplified with PCR before being spotted on NALFIA strips. This technique had a sensitivity of 98% and a specificity of 99% (PCR as a gold standard). The limit of detection was measured by using serial dilutions of a *Plasmodium* culture and was 0.3 –3 parasites/μL. The NALFIA is one of the best suited NATs for use in POC settings because it is extremely fast, has high sensitivity and specificity, is simple to use, and the result is determined through visual detection. Some of the drawbacks of NALFIA are that it requires a separate amplification step, and that it includes antibodies similar to those used in RDTs and therefore would likely have many of the same storage requirements that trouble RDTs.

The major advantages of adapting PCR for POC diagnosis is that it is an extensively tested, proven technique (under laboratory conditions), which is capable of detecting extremely low level infections with high specificity and sensitivity. The drawback to using PCR at the POC is that it requires a large amount of infrastructure, including a thermocycler, a steady power supply, and reagent-grade water.^79^ A variety of novel detection methods have been investigated, which when coupled with PCR, offer the potential for rapid (< 1 hour total time) accurate diagnosis and the potential to require less resources, less user intervention, and be more POC accessible than laboratory PCR. These detection methods are especially promising for future development because some of them may be coupled with isothermal amplification techniques to create totally self-contained tests.

## Conclusions

There is a growing interest in developing NATs for malaria diagnosis at the POC. The greatest advantage of NATs is their ability to detect extremely low level infections, which are often missed by microscopy and RDTs. The greatest barrier to NAT use in the past has been their relatively high cost and the amount of infrastructure required. These problems have been addressed in a variety of ways to decrease the cost, increase the ease of use, and maintain a high sensitivity and specificity and low limit of detection. The degree to which current and emerging malaria diagnostics have been able to meet the requirements of an effective POC diagnostic is summarized in Table 3. For the foreseeable future, NATs will likely remain more expensive for malaria diagnosis than RDTs, but may provide significant performance benefits for certain situations, which might outweigh their increased costs.

Isothermal amplification techniques, can be run in a short timeframe, require minimal infrastructure, and can detect extremely low levels of infection with high accuracy. The LAMP, in particular, is promising for further development as POC tool because it enables easy detection of the amplified product, is reasonably inexpensive, and it can be operated with minimal sample preparation. The greatest drawback for isothermal amplification is that, so far, it is relatively untested in realistic field conditions, which might significantly impact the performance of these tests.

The POC-focused detection strategies for more conventionally amplified targets generally focus on reducing the per test cost with methods that enable extremely high throughput, such as single-pot speciation. Although these techniques can be extremely sensitive, quick, and relatively inexpensive, many of them may be difficult to adapt into POC tools because of their reliance on extensive infrastructure. The NALFIA is the detection technique that may be best suited to the POC because it has already been tested under field conditions and is quick and simple to use. In addition, future work coupling NALFIA with an isothermal amplification system could result in a diagnostic that requires little infrastructure and maintains a low limit of detection compared with microscopy and RDTs.

Overall, the NATs being developed for detection of malaria at the POC have demonstrated that NATs can be easy to perform and maintain a low limit of detection and high sensitivity and specificity. The use of LAMP as a low-cost amplification method, coupled with low-cost detection such as turbidity or a scheme such as the PCR LDA, have the potential to create an NAT that is accessible in POC environments. The next major step in NAT development will be testing these techniques under realistic field conditions in malaria-endemic countries. The integration of POC appropriate amplification technologies such as LAMP and NASBA with low-cost and easy to use detection systems such as NALFIA will be an important next step in realizing this goal.

## Figures and Tables

**Table 1:** Summary of diagnostic performance of established malaria diagnostics*

Assay	Reference	*Plasmodium* species detected	Limit of detection	Sensitivity (gold standard)	Specificity (gold standard)
Laboratory-based thin blood smear microscopy	19	*falciparum*, *vivax*, *malariae*, *ovale*	50 parasites/μL	95.7% (consensus of microscopy and PCR)	97.9% (consensus of microscopy and PCR)
Field-based thin blood smear microscopy	21	*falciparum*, *vivax*, *malariae*, *ovale*	50 parasites/μL	10% (laboratory microscopy)	99.3% (laboratory microscopy)
RDTs tested in malaria-endemic countries	31	*falciparum*, *vivax*	> 100 parasites/μL	80–95%(microscopy)	85% (microscopy)
Laboratory PCR	43	*falciparum*, *vivax*, *malariae*, *ovale*	0.5–5 parasites/μL	100%†	100%†

* RDTs = rapid diagnostic tests.

† Laboratory polymerase chain reaction (PCR) is generally considered the most sensitive of the established diagnostics for malaria and is used as the gold standard when comparing it to other malaria diagnostic techniques.

**Table 2:** Summary of diagnostic performance of potential point-of-care appropriate nucleic acid tests*

Assay	Reference	*Plasmodium* species detected	Limit of detection	Sensitivity (gold standard)	Specificity (gold standard)
LightCycler PCR	65	*falciparum*, *vivax*, *malariae*, *ovale*	10 parasites/μL	97% (microscopy)	100% (microscopy)
RT-PCR	67	*falciparum*, *vivax*, *malariae*, *ovale*	< 0.1 parasites/μL	100% (microscopy)	93% (microscopy)
PCR LDA	66	*falciparum*, *vivax*, *malariae*, *ovale*	1 parasite/μL	100% (PCR)	90% (PCR)
	68		–10 parasites/μL		
PCR ELISA	70	*falciparum*, *vivax*, *malariae*, *ovale*	< 30 parasites/μL	91.4% (microscopy)	95.8% (microscopy)
LAMP	57	Pan, *falciparum*, *vivax*, *malariae*, *ovale*	0.2 parasites/μL (*malariae*, *ovale*), 2 parasites/μL (Pan, *falciparum*, *vivax*)	98.5% (microscopy)	94.3% (microscopy)
	56	*falciparum*	≥ 0.2 parasites/μL	95% (PCR)	99% (PCR)
	58	*falciparum*		76.1% (PCR)	89.6% (PCR)
	60	Pan, *falciparum*	5 parasites /μL	93.3% (PCR)	100% (PCR)
NASBA	38	*falciparum*	0.02 parasites/μL	100% (microscopy)	86% (microscopy)
	62	*falciparum*, *vivax*, *malariae*, *ovale*	0.01–0.1 parasites/μL		
NALFIA	71	Pan	0.3–3 parasites/μL	98% (PCR)	99% (PCR)

* PCR = polymerase chain reaction; RT = reverse transcription; LDA = lactate dehydrogenase assay; ELISA = enzyme-linked immunosorbent assay; LAMP = loop-mediated isothermal amplification; NASBA = nucleic acid sequence–based amplification; NALFIA = nucleic acid lateral flow immunoassay.

**Table 3:** Comparison of diagnostic characteristics that are relevant to the ability of a test to be useful at the point-of-care*

Assay	Reference	Limit of detection	Time	Cost/test	Requirements	Tested in field?
Microscopy	19, 21	50 parasites/μL	20 minutes/slide	$0.20†	Trained personnel, microscope, Giemsa stain	Yes
RDT	31	> 100 parasites/μL	20 minutes	$0.45–$1.40†	Cold chain for storage/transport of RDTs	Yes
Laboratory-based PCR	43	< 5 parasites/μL	1 hour	$1.50–$4 (reagents only)	Themocycler, cold chain, power, reagent grade water	No
RT-PCR	65, 67	0.1–10 parasites/μL	1 hour	$4–$5 (reagents only)	DNA extraction, thermocycler, reagent water, power	No
PCR LDA	66, 68	0.3–10 parasites/μL		$0.30 (detection only)	DNA extraction, PCR	No
PCR ELISA	70	< 30 parasites/μL	6 hours		DNA extraction, heat source, wellplate reader	Yes (Army hospital in Thailand)
LAMP	56, 57, 58, 60	0.2–5 parasites/μL	30 minutes–2 hours	$0.40–$0.70 (reagents only)	Heat source for amplification/DNA extraction,	No
NASBA	38, 62	0.01–0.1 parasites/μL	60 minutes	$5–$20 (reagents only)	Heat source for amplification, RNA extraction method, fluorescence measurement system	No
NALFIA	71	0.3–3 parasites/μL	1–1.5 hours		Self-contained test	Yes (Mbita, Kenya)

* RDT = rapid diagnostic test; PCR = polymerase chain reaction; RT = reverse transcription; LDA = lactate dehydrogenase assay; ELISA = enzyme-linked immunosorbent assay; LAMP = loop-mediated isothermal amplification; NASBA = nucleic acid sequence–based amplification; NALFIA = nucleic acid lateral flow immunoassay.

† Costs listed for more established techniques (microscopy PCR and RDTs) reflect a more realistic cost to the end user including shipping, storage, and reagent costs. Costs listed for the techniques under development (RT-PCR, PCR LDA, PCR ELISA, LAMP, NASBA, and NALFIA) reflect only the cost of the materials required to perform the assay as given by the authors of the cited studies and likely underestimate the ultimate end cost to the user.
